# Supplementary material for: Optical skyrmions from metafibers with subwavelength features
Source: Nat Commun. 2024 Nov 22;15:10141. doi: 10.1038/s41467-024-54207-z (PMC11584664; doi:10.1038/s41467-024-54207-z)
Supplement: Supplementary file 1 — Supplemetary Information [file 41467_2024_54207_MOESM1_ESM.pdf]

Optical skyrmions from metafibers with subwavelength features: Supplementary Material.

Tiantian He,[1,2,†] Yuan Meng,[1,3,†] Lele Wang,[1,2] Hongkun Zhong,[1,2] Nilo Mata-Cervera,[4] Dan Li,[1,2] Ping Yan,[1,2] Qiang Liu,[1,2] Yijie Shen[4,5,*] & Qirong Xiao[1,2,*]

[1] *Department of Precision Instrument, Tsinghua University, Beijing 100084, China*

[2] *State Key Laboratory of Precision Space-time Information Sensing Technology, No.1 Qinghua Garden, Chengfu Road, Beijing 100084, China*

[3] *Present Address: Mechanical Engineering and Materials Science, Washington University in St Louis, St Louis, MO 63130, USA*

[4] *Centre for Disruptive Photonic Technologies, School of Physical and Mathematical Sciences & The Photonics Institute, Nanyang Technological University, Singapore 637371, Singapore*

[5] *School of Electrical and Electronic Engineering, Nanyang Technological University, Singapore 639798, Singapore*

[†] Equal Contribution

[*] E-mails: yijie.shen@ntu.edu.sg, xiaoqirong@mail.tsinghua.edu.cn

Supplementary Note 1: The design strategy of metasurface

In cylindrical coordinates(r, ϕ, z), the complex amplitude of the nth-order Bessel beam (BB) propagating along the z-axis can be described by[1]

$$E(r, \phi, z) = AJ_n(K_r r)\exp(ik_z z)\exp(\pm in\phi) \quad \text{(S1)}$$

where A is the amplitude, J_n is the *n*th Bessel function of the first kind, $k = \sqrt{k_r^2 + k_z^2}$ is the total wavenumber, while k_z and k_r are the transverse and longitudinal wave numbers, ϕ is the azimuthal angle, which represents the high-order BBs (n ≠ 0) must carry orbital momentum and have zero intensity along z-axis at $r = 0$.

Although the generation of ideal BBs requires an infinite amount of power, they can be greatly approximated using a uniform illumination over an axicon, which can be achieved in real settings. On the other hand, BBs carrying topological charge ($\ell \neq 0$) have a helical wavefront, which can be generated by propagating a flat wavefront through both an axicon and a spiral phase plate. That is, the phasefront of J_1 can be obtained just by superimposing a vortex phase on J_0. The order of the BB ℓ is determined by the topological charge induced by the spiral phase plate, and its amplitude profile can be controlled by changing the parameters of the axion. In addition, the beam in the expansion section diffuses, resulting in an additional spherical phase on the fiber facet. To correct the resulting wavefront, a correction phase $-P_S(x, y)$ is superimposed to the design phase, compensating the wavefront $P_S(x, y)$. To determine P_S, we obtain the field complex amplitude field distribution after propagating at a certain distance using the simulation in the Supplementary Note 2.

Therefore, the target phase profiles of dual-polarizations metasurface[2] can be expressed as:

$$\phi_x = -P_s + \frac{2\pi}{\lambda}\sqrt{x^2+y^2}\mathrm{NA}_1 \quad \text{(S2)}$$

$$\phi_y = -P_s + \frac{2\pi}{\lambda}\sqrt{x^2+y^2}\mathrm{NA}_2 + \phi_s \quad \text{(S3)}$$

where λ is the working wavelength centered at 1.55 μm, (x, y) indicates the position of each nanoantenna, NA refers to the numerical aperture of the BB, $\phi_s = \arctan(y/x)$ is the azimuthal coordinate, associated with the phase profile of an $\ell = 1$ vortex. The phase profiles are illustrated in Fig. S1(a). The first row shows the phase of J_0 (C) polarized along the x-axis, while the second row shows the phase of J_1 (E), polarized along y-axis. The combination of the fiber's correction phase $\phi_{\mathrm{A}} = -P_s(x, y)$ and the axion phase ϕ_{B} generates the J_0 phase profile ϕ_{C}. The addition of the helical phase ϕ_{D} into the J_0 phase ϕ_{C} yields the first-order Bessel phase profile ϕ_{E}. To realize skyrmions with subwavelength polarization features, both NA_1 and NA_2 are chosen as 0.8.

The metasurface is composed by arrays of meta-units illustrated in Fig. S1(b), consisting of amorphous silicon (a-Si) antennas laying on top of a SiO_2 substrate. Due to the high refractive index contrast between the a-Si (n = 3.48) and air (n = 1), these nanoantennas act as a birefringent waveguides[3], with very high transmissivity and negligible cross-talk. Following the propagation phase method, the geometries of the nanopillars map different wavefronts in the x and y polarizations as in Eq. (S2-S3). To achieve optimal performance, the parameters D_x and D_y of the mth antenna are determined using the following optimization formula[2]:

$$\min_{(D_x,D_y),\phi_0\in[0,2\pi]} \left\{\sum_m \max\left\{\left|\phi_x^{design} - \phi_x^{simulation}\right|, \left|\phi_y^{design} - \phi_y^{simulation}\right|\right\}\right\} \quad \text{(S4)}$$

where ϕ_x and ϕ_y represent the phase changes of the incident electric field polarized along x- and y- axes respectively. The nanoantenna responses are numerically calculated sweeping all the combinations of D_x and D_y. Considering the implications of the system's two-fold (C_2) rotational symmetry[4], the phase shifts $\phi_y/2\pi$ and transmissivity T_y in the y polarization can be easily obtained by swapping the x and y axes of the x polarization response. In this way, different functionalities are achieved in orthogonal polarizations, so that the dual-order BBs generators can be applied in fiber-facet integrated with a single-layer metasurface.

Due to the Nyquist sampling theorem, at least two discretization elements must be placed within the 2π phase coverage to produce BBs with a large NA and subwavelength resolution. In order to meet the phase requirement and for ease of fabrication, the square lattice period is fixed at P = 700 nm, much smaller than the wavelength to minimize diffracting effects. The antennas' height H is fixed as 900 nm while the length D_x and D_y of the principal axes x and y change from 250 to 550 nm to modulate ϕ_x and ϕ_y. Fig. S1(c) and (d) show the phase shift $\phi_x/2\pi$ and transmissivity T_x of the unit cell when shined with x-polarized light at the working wavelength λ = 1.55 μm. The phase shift for y polarization can be obtained by transposing the phase shift in x polarization. The results are obtained with vector simulations with the commercial software Lumerical FDTD solutions. In the unit cell, periodic boundary conditions are applied in the x and y directions and perfectly matched layers are applied in the z direction.

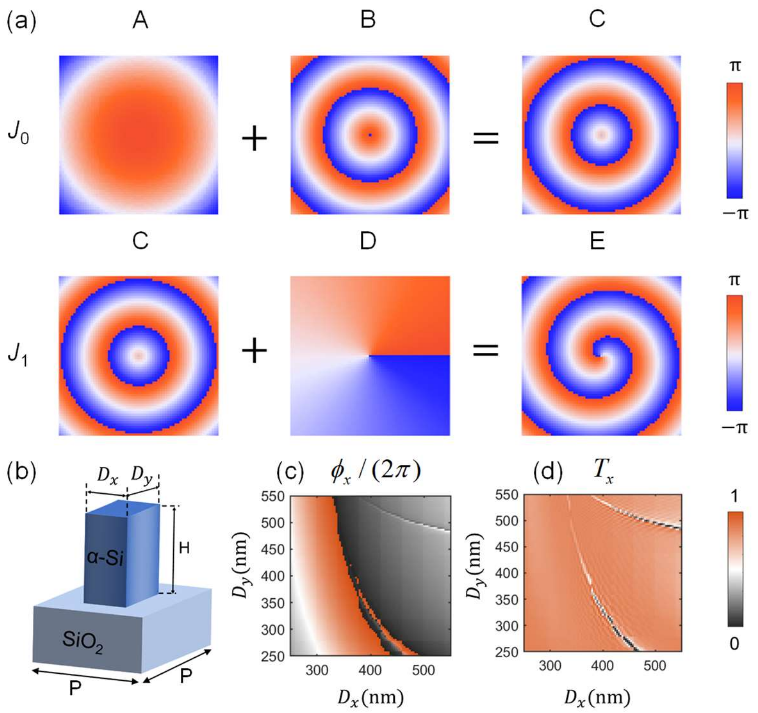

Fig. S1 (a) The phase distributions superimposed on the dielectric metasurface are denoted by A, B, C, D, E, which correspond to the correction phase of the source, the axion phase, the phase of J_0, the phase of helical vortex, the phase of J_1. (b) The unit cell of the metasurface. (c) Phase shift and (d) Transmissivity for *x* polarization.

Supplementary Note 2: Diffusion analysis of light within expansion section and chosen light distribution

The PSF (Polarization Maintaining Fiber, PM1550-HP) has a core diameter of 8.5 μm and a cladding diameter of 125 μm. Light coming out of the PSF enters the expansion section and diffuses. The light beam expands as it propagates, as shown in Fig. S2. Typically, the size of the metasurface is larger than the size of the light field in order to ensure high light utilization efficiency. However, large metasurface sizes lead to higher fabrication costs. If the radius of the metasurface becomes m times larger, the area becomes m^2 times larger, resulting in m^2 times the processing cost. Knowing that the full width at half maximum contains most of the energy of the light field, designing the metasurface diameter slightly larger than the full-width half-maximum of the input field maximizes the trade-off between high efficiency and low cost. Therefore, considering a metasurface diameter of 56 μm, we choose a propagation distance of 350 μm, so that the FWHM of the input light field is 43 μm. The intensity and phase distributions are plotted in Fig. S2(c) for both an XY cross section and an *x*-axis slice. In addition, the expansion section is composed of two parts: the SiO_2 substrate of the metasurface and the ultraviolet curing glue which links the fiber holder and the metasurface substrate, and they have thickness of 300 μm and 50 μm, respectively. This refractive index of the glue matches that of the quartz substrate and the fiber cladding 1.44.

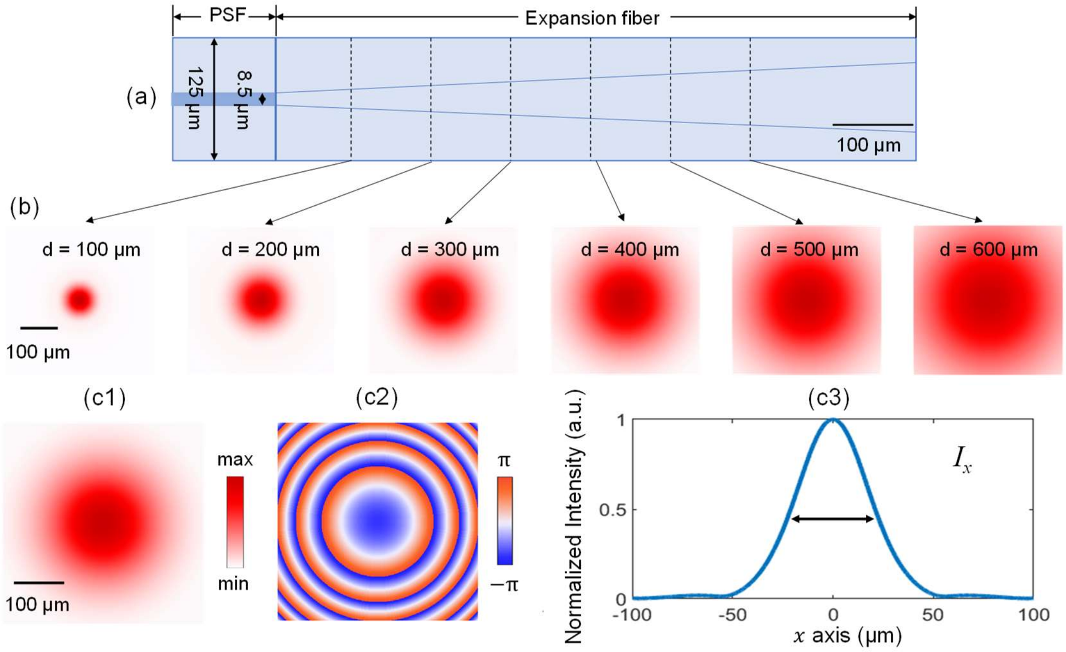

Fig. S2 Analysis of light propagation within expansion section. The index of expansion section is same to SiO_2. (a) Cross-sectional view of optical fiber along the propagation direction. (b) The transverse light field distributions in different propagating distances. (c1-c2) The complex amplitude distribution and (c3) intensity curve of selected light source.

Supplementary Note 3: The skyrmion density and skyrmion number in regulation

The Stokes parameters of skyrmion are reconstructed from the relations as:

$$\begin{pmatrix} S_0 \\ S_x \\ S_y \\ S_z \end{pmatrix} = \frac{1}{E_R^2 + E_L^2} \begin{pmatrix} E_R^2 + E_L^2 \\ 2 \cdot E_R \cdot E_L \cdot \cos(\varphi_R - \varphi_L) \\ 2 \cdot E_R \cdot E_L \cdot \sin(\varphi_R - \varphi_L) \\ E_R^2 - E_L^2 \end{pmatrix} \tag{S5}$$

where E_R and E_L represent the recovery complex amplitude of the RCP and LCP components. The skyrmion density is calculated by Eq. (3) and its integration over the transverse plane yields the skyrmion number as in Eq. (2). To characterize the mode evolution from non-skyrmion to skyrmion, the skyrmion densities of different polarization angles are illustrated in Fig. S4. When the polarization angle is equals to 0°, the light profile corresponds to the scalar zeroth-order BB, showing a zero skyrmion number; in the same way as when angle equals to 90°, the profile is a pure scalar first-order BB, showing again a near-zero skyrmion number. When the angle varies between 40°-70°, the intensities of zeroth-order and first-order BBs are comparable one to each other, showing a stable and skyrmion with near unity skyrmion number. The PS coverage along θ is also illustrated in Fig. S4. As we can see, between 40° to 70°, the skyrmion numbers are extremely close to 1, proving that our skyrmion generator is capable of exciting a nearly perfect skyrmion at angles larger than 30°. This means that in practice, the polarization of the light source does not need to be adjusted very precisely to achieve the desired skyrmion, which is very useful in practical applications.

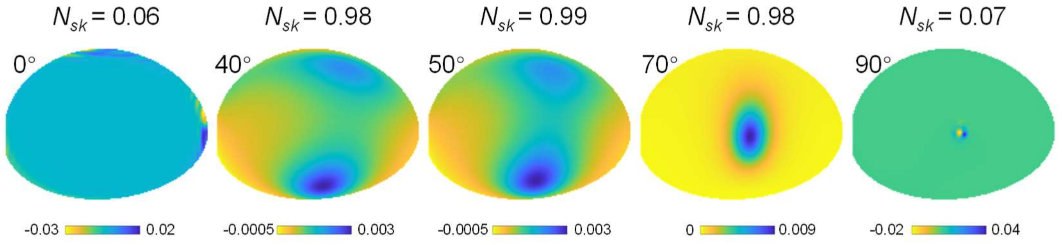

Fig. S3 The skyrmion densities for different input polarization angles.

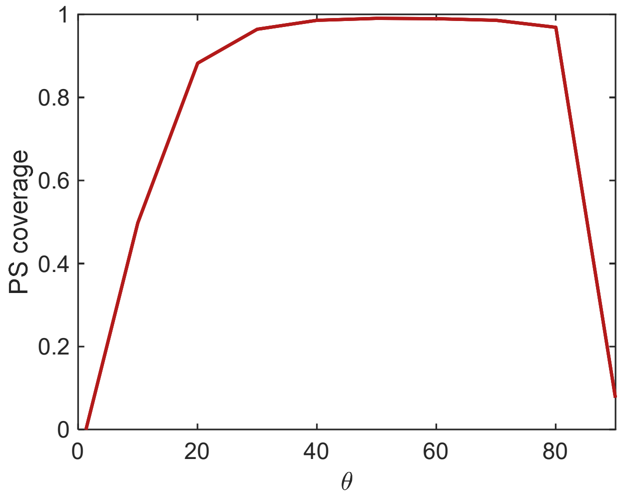

Fig. S4 The Poincaré sphere (PS) coverage as a function of the polarization angle θ.

Supplementary Note 4: The Poincaré Sphere in regulation

A more detailed description of the skyrmion type using the Poincaré Sphere with different polarization angles θ at intervals of 10° is illustrated in Fig. S5. A Stokes skyrmion can be realized when the HL-colored map covers the entire sphere. As seen in the figure, the HL are almost fully mapped when θ ranges from 40° - 70°, showing high quality skyrmions. Meanwhile, the interconversion between skyrmion and bimeron can be realized by a quarter-wave plate, whose effect is equivalent to a transformation of the polarization basis, which corresponds to the transformation of the coordinate axis of the Poincaré sphere. To take a special case, when the angle between the fast-axis of quarter-wave plate and the x-axis is 45°, the coordinate system of the Poincaré sphere (S_x,S_y,S_z) changes to (S_z,S_x,S_y). This coordinate transformation does not affect the topology as well as the skyrmion number, but causes a transformation of the Stokes field, as specified in Methods. The bimeron type is also represented in the Poincaré Sphere in Fig. S6.

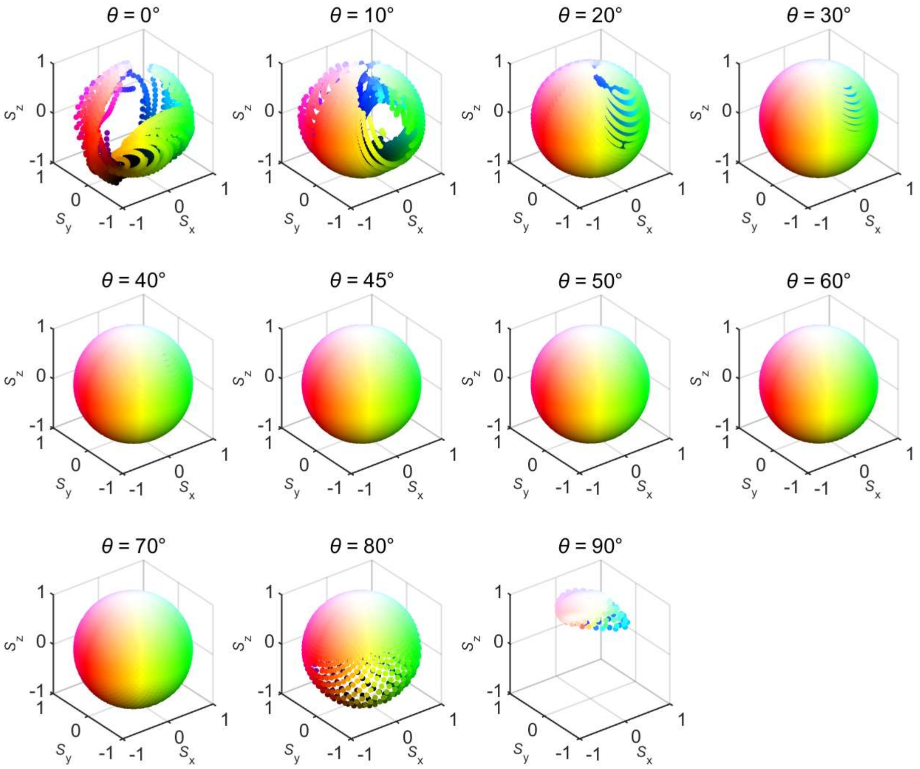

Fig. S5 The Poincaré Sphere in simulations when the polarization angle θ changes: transitions between skyrmion and non-skyrmion states.

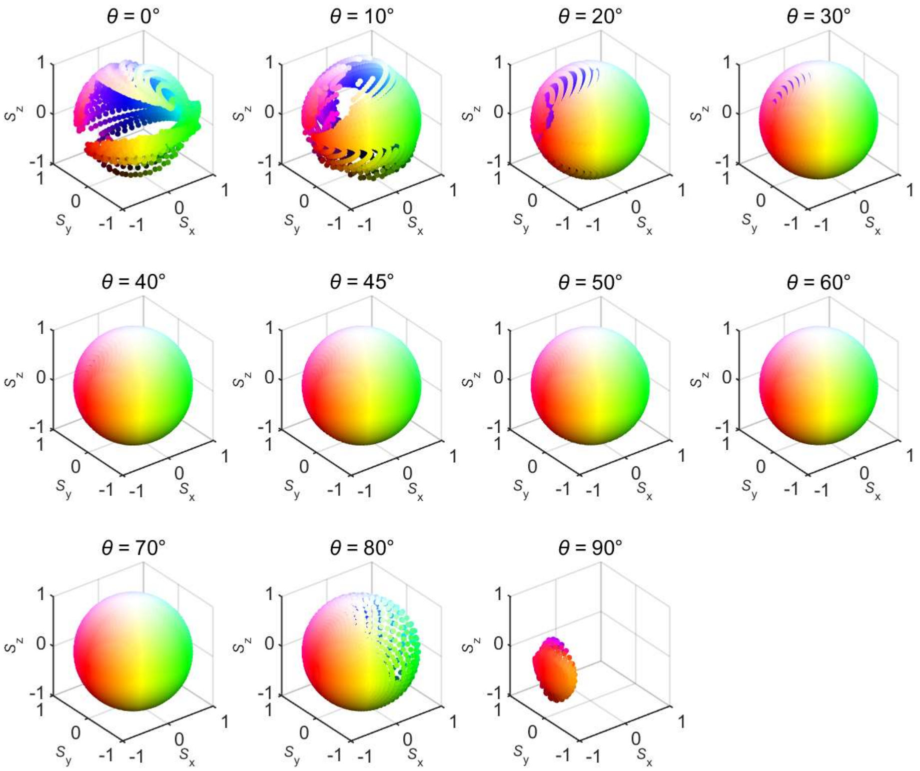

Fig. S6 Simulation of the Poincaré Sphere when changing the polarization angle θ: transformation between bimeron and non-bimeron states.

Supplementary Note 5: The broadband and non-diffraction properties of proposed device

The broadband properties of our proposed device are also demonstrated. The profiles of J_0 and J_1 and their corresponding vector fields for different wavelengths in the range of 1.45 μm to 1.65 μm are shown in Fig. S7(a-b). It is worth noting that the difference in refractive indices of the a-Si and SiO_2 as well as the difference in the size of the light source after the expansion section for different wavelengths are also taken into account in the simulations. As we can see, the profiles of J_0 and J_1 experience subtle changes, while the Stokes vector fields show a highly robustness over the 200 nm bandwidth.

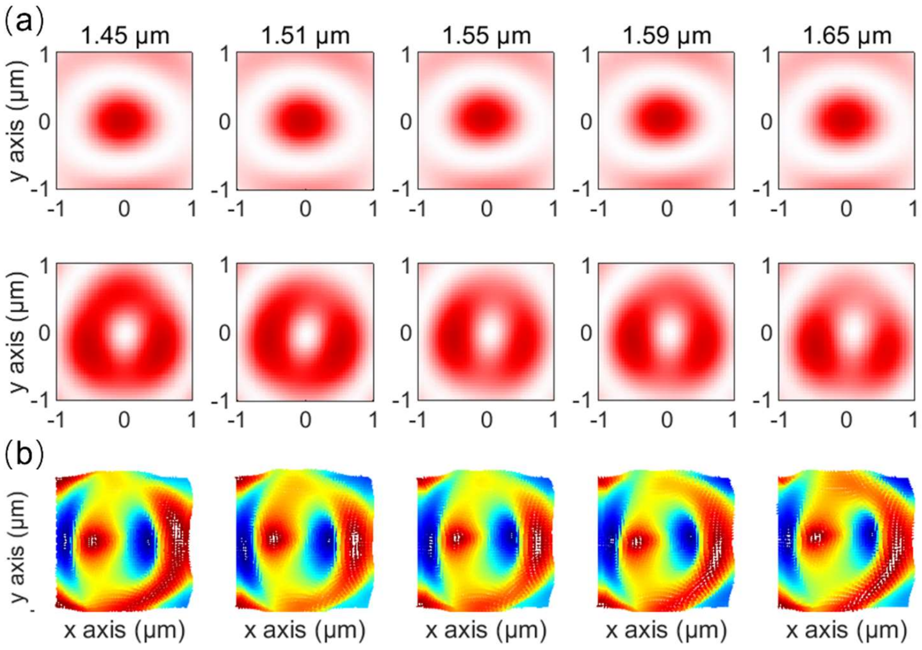

Fig. S7 (a) Measured intensity distributions of the *x*-polarization (upper row) and *y*-polarization (lower row) from 1.45 μm to 1.65 μm respectively. (b) The corresponding vector distributions over the 200 nm bandwidth.

Besides, we have also simulated the construction of Stokes skyrmion over different distances in the depth of focus of quasi-Bessel beams. The intensity distributions under orthogonal polarizations and the vector distributions are shown in Fig. S8. And the Stokes Poincaré spheres over different propagation distances and corresponding skyrmion numbers are also illustrated in Fig. S9. It can be observed that as the propagation distance increases, the transverse electric field distributions and vector distributions of the realized Stokes skyrmion, as well as the Stokes numbers, remain nearly unchanged, demonstrating a nearly non-diffraction property. It indicates that the presence of longitudinal electric component E_z seldom impacts the transverse polarization distribution, barely disrupting the topological features of the Stokes skyrmions reported in this manuscript.

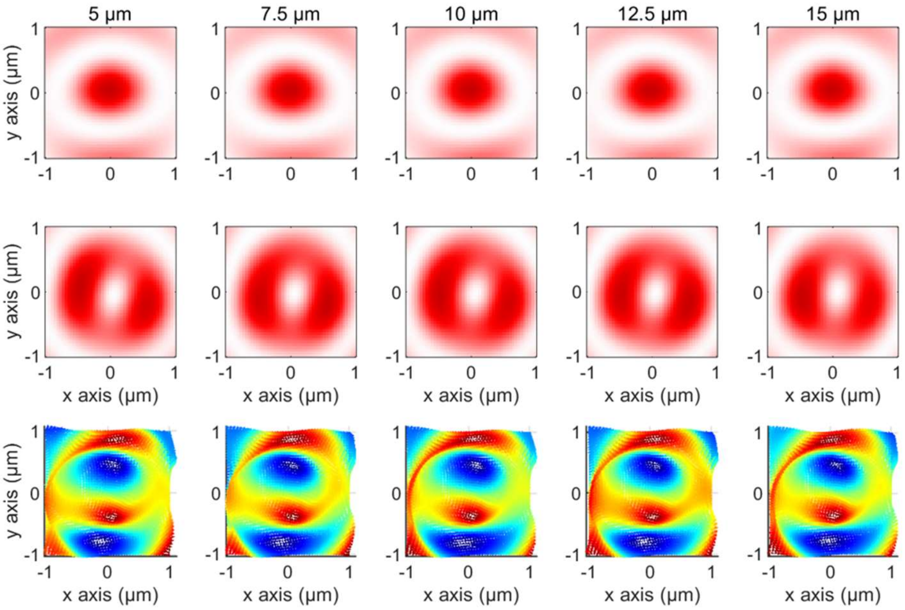

Fig. S8 (a) Measured intensity distributions of the *x*-polarization (upper row) and *y*-polarization (middle row), and vector distributions (lower row) after different beam propagation distances from 5 μm to 15 μm with a step of 2.5 μm accordingly.

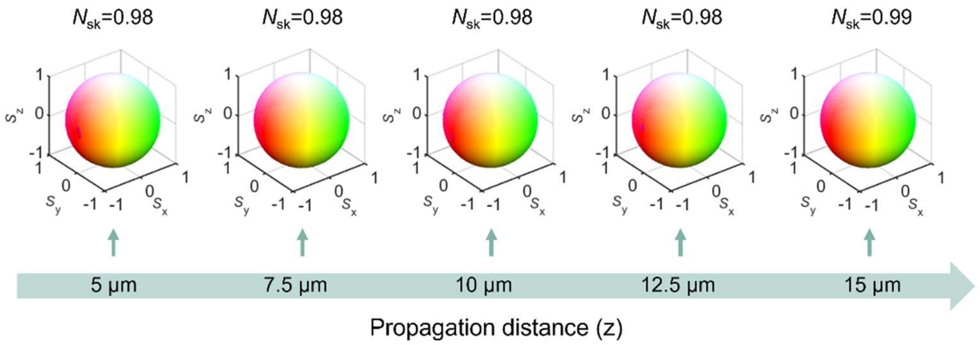

Fig. S9 The corresponding Stokes Poincaré spheres with skyrmion numbers shown over different propagation distances off the fiber facet.

Supplementary Note 6: Fabrication procedure

To ensure the high-precision tuning of the propagation phase, we selecte EBL technology for the metasurface fabrication. The flowchart for fabrication procedure is shown in Fig. S10. In the figure, gray represents quartz substrate, gold represents a-Si, light yellow represents photoresist, and black represents Cr.

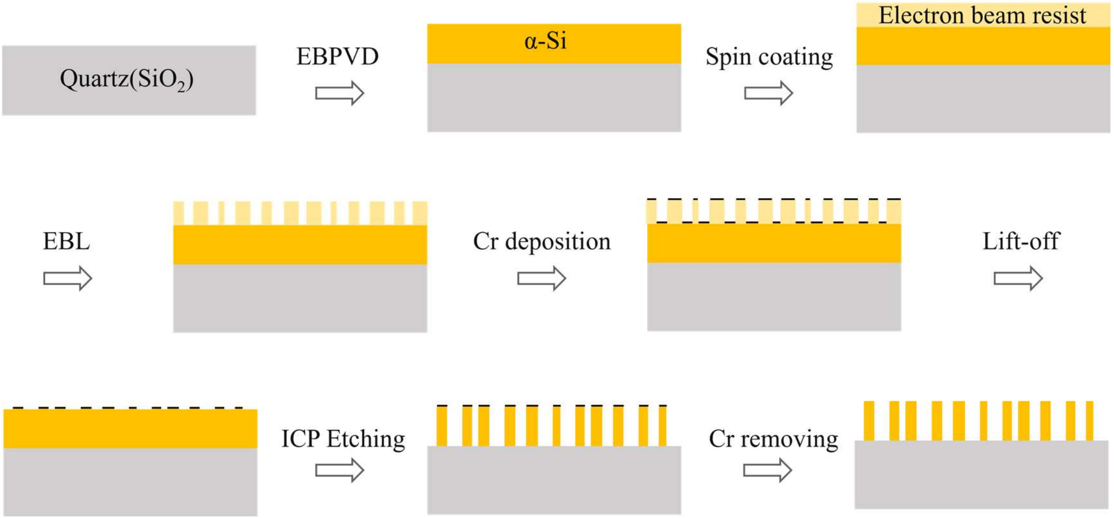

Fig. S10 Illustration of the metasurface fabrication procedure. EBPVD: Electron Beam Physical Vapor Deposition. EBL: Electron Beam Lithography. ICP Etching: Inductively Coupled Plasma Etching.

Supplementary Note 7: Analysis of errors in fabrication procedures and experimental processes

The two most significant errors in the proposed metafiber may occur from the fabrication procedures and the experimental processes. Dimensional errors during fabrication can result in variations in the size and shape of the structure that can affect its performance. Misalignment between the fiber center and the metasurface center can lead to distortion of the optical signal, and an angle deviation between the optical axis of the fiber and the normal direction of the metasurface could cause signal distortion. Therefore, a thorough analysis of these errors is essential to ensure the robustness and reliability of the structure.

Considering the resolution of the fabrication methods like EBL and ICP, fabrication error may induce imprecisions in the height and size of the antennas. Firstly, we perform an error analysis with respect to the height of the antenna. The designed height is 0.9 μm, so we analyze two heights, 0.85 μm and 0.95 μm to study the performance robustness of the device. The profiles J_0 and J_1, as shown in Fig. S11, indicate a very slight diffusion the and a small increase sizes, but the overall distributions remain consistent, demonstrating good robustness. Secondly, size errors typically include consistent errors and random Gaussian errors. The upper two rows of Fig. S12 depict the output fields for a metasurface subjected to consistent size error of 5 nm, 10 nm, and 15 nm. The lower two rows of Fig. S12 display the profiles when antennas are subjected to gaussian errors with the variance of 5 nm, 10 nm, 15 nm. The black dashed line shows the ideal results, and we can see that when the errors are introduced, the intensity distributions of J_0 and J_1 remain almost unchanged, demonstrating excellent robustness.

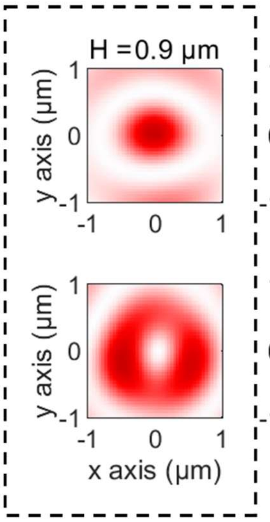

Fig. S11 The analysis of antennas height error. The three columns depict the intensity profile over height of 0.9 μm, 0.85 μm, 0.95 μm, from left to right respectively. The upper and lower rows represent profile in *x* and *y* polarizations respectively. The black box indicates our design's profiles.

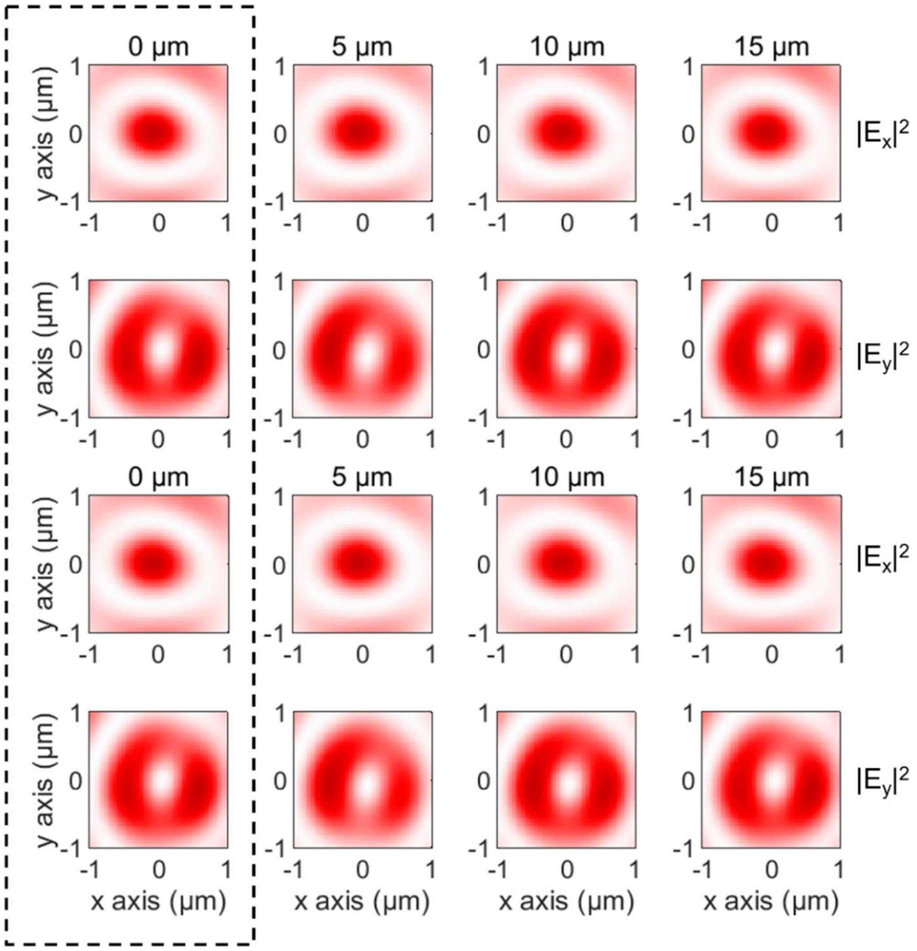

Fig. S12 The analysis of antennas size error. The upper two rows depict the intensity profile over consistent errors of 5 nm, 10 nm, 15 nm in the *x* and *y* polarizations respectively. The third row and the fourth row depict the intensity profile over gaussian errors with the variance of 5 nm, 10 nm, 15 nm in the *x* and *y* polarizations respectively. The black box indicates our design's profiles.

In the experimental process, it is crucial to align the fiber with the metasurface, which can significantly impact the device's output. The most common errors in this aspect are the misalignment of the fiber optic axis with the vertical axis of the metasurface and the differences in the lateral positions of the two centers, respectively. The illustrations of the angle error are shown in Fig. S13. Ideally, the central optical fiber axis overlaps with the central axis of the metasurface, shown in Fig. S13(a). An indication of angular errors on the x-axis and on the y-axis as shown in Fig. S13(b) and (c), respectively. The left and right columns represent the output profiles when $angle_y = -1^{\circ}$ and $angle_y = 1^{\circ}$, respectively. Then, we add $angle_x = \pm 1^{\circ}$ to the design, so that the profiles shift on the x-axes. If non-zero $angle_y$ appears, the profile will be shifted on the y-axis. Nevertheless, the intensity profile still mimics clear zeroth-order and first-order BBs. The scenarios of lateral positional errors are shown in Fig. S14, showing the mismatch between the fiber center and the metasurface center when we add a ±5 μm lateral positional error. The output optical fields experience a lateral shift, but the overall profile remains stable, indicating that the proposed structure possesses strong robustness.

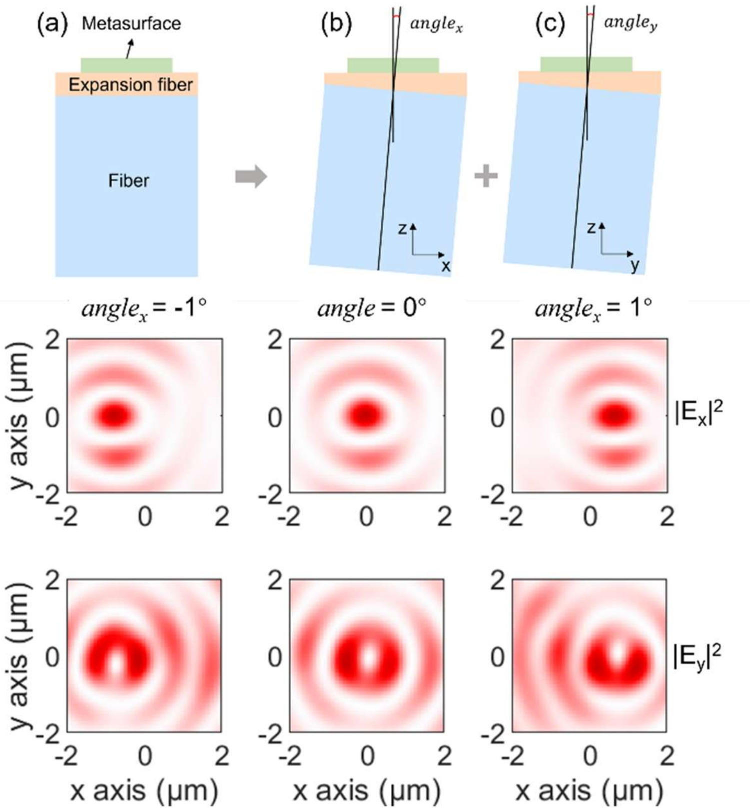

Fig. S13 The error analysis of the angle error. (a)-(c) The illustrations of the angle error.

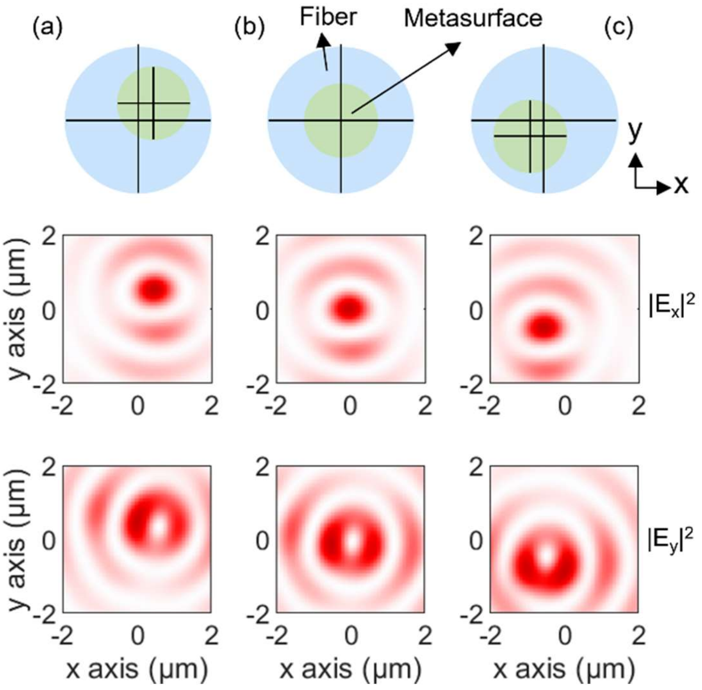

Fig. S14 The error analysis of the lateral positions. Ideally, the two centers are overlapped, shown in (b). Schematics with the center of the metasurface at the lower right and upper left corners of the fiber center are shown in (a) and (c), with its light field below the schematic. The position error steps are chosen as 5 μm in *x* and *y* axis. From the profiles, the light field distributions will be shifted when the position error exists. The distribution of the light field moves in the same direction as the center of the metasurface. Nevertheless, the light field's intensity distribution is almost unchanged, showing outstanding robustness.

Supplementary Note 8: Experimental setup

The experimental setup consists of two optical paths: the amplified light path (upper path) and the coherent light path (lower path). The object wave propagates through the amplified light path, while the collimated plane wave propagates through the coherent light path. The two beams interfere when passing through a polarization beam splitter (PBS), resulting in an interference light field. When both optical paths are simultaneously combined, the intensity distribution resulting from the interference between the object wave and the plane wave can be obtained. By removing the amplified light path, the intensity distribution of the collimated plane wave can be obtained. The polarizers are used in the two paths to select a certain polarization. By changing the directions of the polarizers in *x*- or *y*- axis at the same time, the intensities of interference light fields and collimated plane wave under *x* and *y* polarizations could be collected for complex amplitude recovery in Supplementary Note 9.

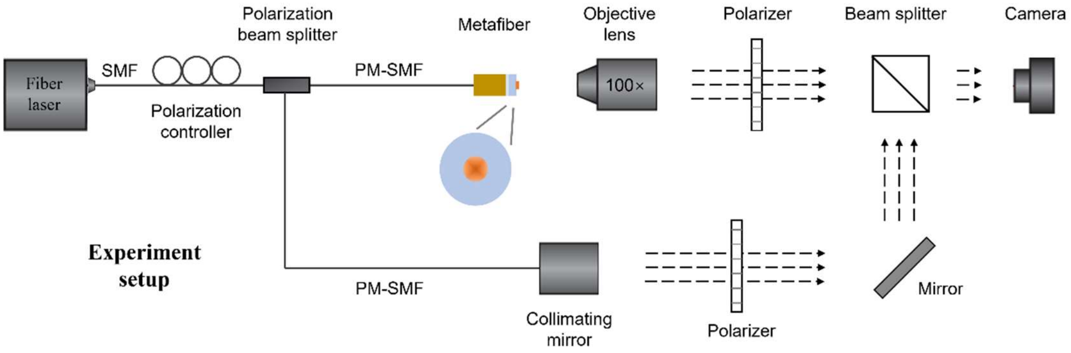

Fig. S15 Experiment setup, containing two optical paths: the amplified light path (upper) and the coherent light path (lower).

Supplementary Note 9: Experimental complex amplitude recovery

From experiment setup in Supplementary Note 8, we could obtain the intensity profiles of inteference waves under *x* and *y* polarizations. The complex amplitude of inteference wave can be obtained from intensity profiles of inteference waves using the procedure shown in Fig. S16. The intensity profiles of plane wave are also obtained in the experience and the phase profiles are planer. The complex amplitude profiles of object wave can be derived by dividing the complex amplitude of inteference wave by the conjugated reference plane wave (shown as Fig. S17 and Fig. S18).

The procedure of recovering the complex amplitude of the inteference fields are illustrated in Fig. S16. Firstly, the intensities of inteference fields (1st column) in *x* (upper row) and *y* (lower row) directions are collected experimentally. Then, the interferometric intensities are Fourier transformed. The Fourier transformed images (2nd column) consist of three main parts: the zeroth-order containing the background information, the first order +1, and the conjugate order -1. Due to the completely off-axis interference mode, the zero-th order, +1- and -1- orders are completely separated in the spectrum of the interferogram. Subsequently, the phase informations are extracted by spatial filtering the first order (3rd column) and performed inverse Fourier transform, obtaining the complex amplitudes of the inteference profiles(object wave + plane wave), shown as the 4th column.

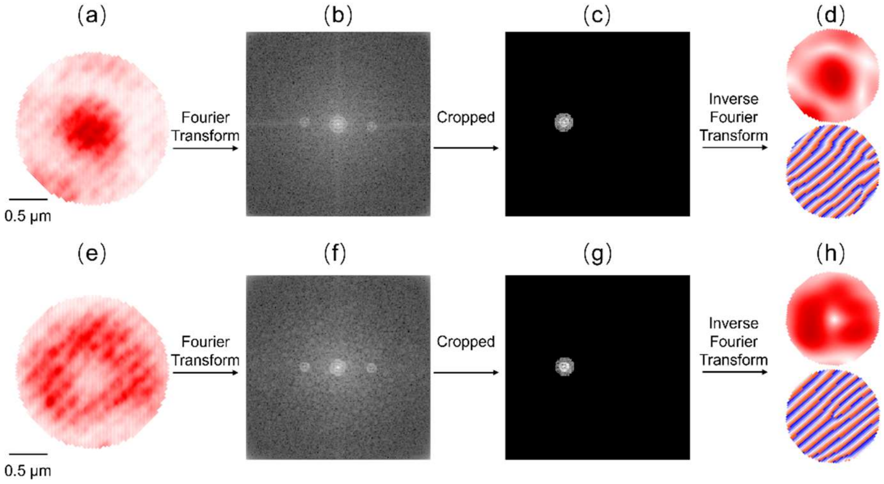

Fig. S16 The process of recovering the complex amplitudes of interference fields under (a)-(d) *x* polarization and (e)-(h) *y* polarization.

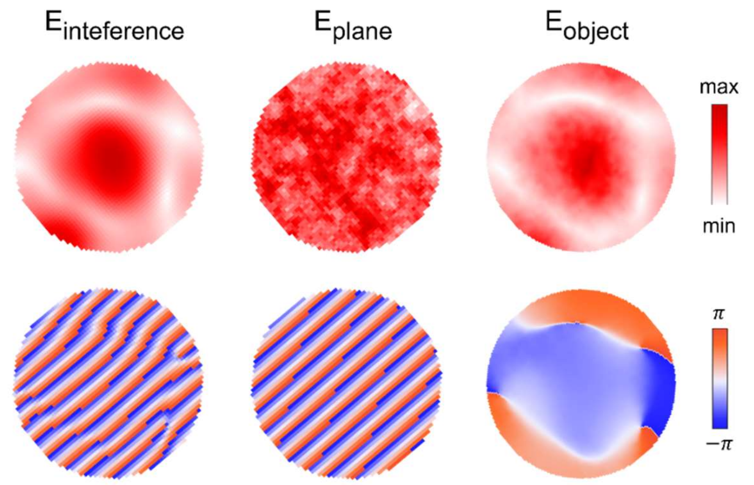

Fig. S17 The process of Complex amplitude recovery in x polarization in experiments. The complex amplitudes of the object wave (third column) are derived by dividing the interference complex amplitude field (first column) by the conjugate complex amplitude profile of the coherent plane beam (second column).

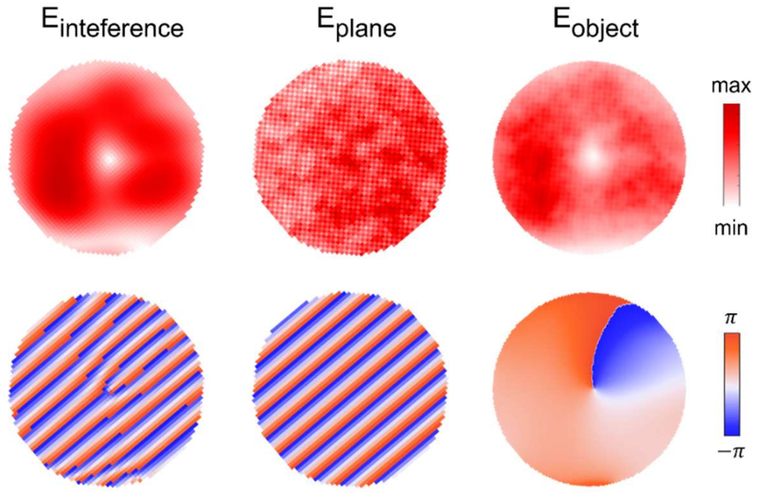

Fig. S18 The process of Complex amplitude recovery in y polarization in experience. The complex amplitudes of object wave (third column) are derived by dividing the interference complex amplitude field (first column) by the conjugate complex amplitude profile of the coherent plane beam (second column).

Supplementary Note 10: The Stokes parameters of skyrmion

Based on the above method, the actual E_x and E_y are obtained, and the corresponding E_L and E_R can be obtained by introducing a quarter-wave plate with the fast axis at 45° to the x-axis, resulting in a Stokes skyrmionic beam. The corresponding experimental Stokes parameters of skyrmion texture calculated by Eq. (S5) are illustrated in Fig. S19.

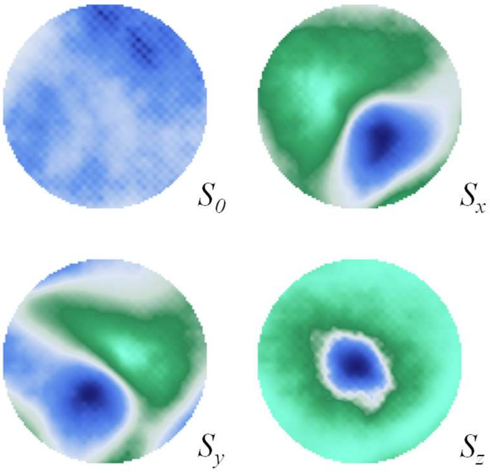

Fig. S19 The polarization distributions of realized skyrmion texture.

Supplementary Note 11: The polarization distribution of bimeron in experiments

Bimeron and skyrmion are two quasiparticles which can be interchanged through a topological transformation using a QWP. By shaping a zeroth-order BB as $|H\rangle$ and first-order as $|V\rangle$, the bimeron state is realized. The polarization distribution of bimeron is also illustrated in Fig. S20. The skyrmion number of bimeron texture calculated from experiments is 0.97, equalling to the skyrmion number of skyrmion texture. For experimentally generated optical skyrmions, the non-integer skyrmion number can be utilized to quantize the quality of the measured skyrmions with respect to the perfect ones.

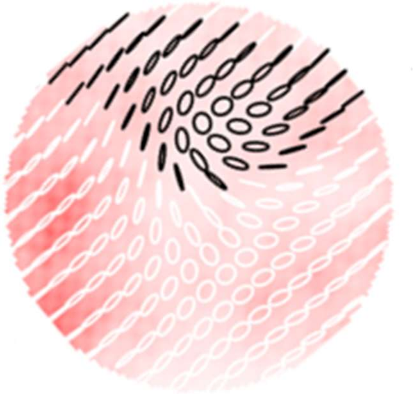

Fig. S20 The polarization distributions of realized bimeron texture.

Supplementary Note 12: The Poincaré Sphere of skyrmion and bimeron in experiments

By means of the Stokes parameters mentioned above, the Poincaré sphere in experiments is illustrated in Fig. S21. The skyrmion and bimeron states can be obtained when the input light source is polarized at 45°, and their Poincaré spheres are shown in Fig. S21b and Fig. S21c. From the figures, the entire HL-colored maps are covered except very few points, showing high quality skyrmions, and they are well consistent with the obtained skyrmion number of 0.97. When the source polarization angle is 0° (Fig. S21(a)), the resulting beam is scalar and only contains a single point in the Poincaré sphere (S_x, S_y, S_z) = (0, 0, 1), which corresponds to the RCP polarization. Similarly,

when the input polarization angle is 90° (Fig. S21(d)), the output polarization is (S_x, S_y, S_z) = (0, 0, -1) , which corresponds to the LCP.

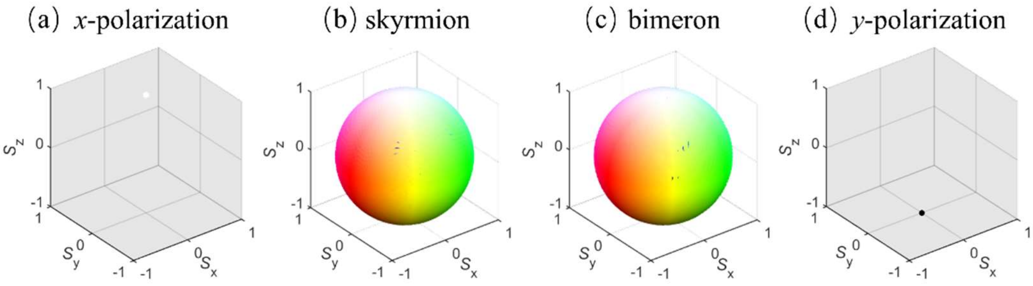

Fig. S21 The Poincaré Sphere of skyrmion, bimeron and non-skyrmion states in experience.

Supplementary Note 13: Error discussions

The simulation results of the electric field are in good agreement with the experimental results, but still have minor variations, mainly from several aspects. Firstly, the size of the generated beams is extremely small with respect to the pixel size of the camera (5.2 μm), leading to a sparse sampling although the captured beams are magnified by 100×. The insufficient resolution may result in the loss of points, causing a slight difference in the intensity profiles. Secondly, the process of complex amplitude recovery is sensitive to misalignment between the two arms of the interferometers, which include the propagation directions as well as polarizers' directions in the two optical paths cannot be perfectly parallel, yielding small errors in the reconstructed complex field. Thirdly, the accuracy of the processing methods could cause discrepancies with respect to the ideal case. After computationally extensive simulations and experiments, considering the constrained processing conditions, the phase shift of 2π, and the optimal illumination scheme, an antenna period of 700 nm with dimensions ranging from 250 to 550 nm was determined. However, deviations are inevitable during metasurface fabrication and experiments. Still, the experimental results verified the great robustness of the device under the different kinds of imperfections. A detailed error analysis the metasurface is presented in the Supplementary Note 6, demonstrating an excellent robustness of our proposed device.

Supplementary References

1 Chen, W. T., Khorasaninejad, M., Zhu, A. Y., Oh, J., Devlin, R. C., Zaidi, A. *et al.* Generation of wavelength-independent subwavelength Bessel beams using metasurfaces. *Light: Science & Applications* **6**, e16259-e16259 (2017).

2 Meng, Y., Liu, Z., Xie, Z., Wang, R., Qi, T., Hu, F. *et al.* Versatile on-chip light coupling and (de)multiplexing from arbitrary polarizations to controlled waveguide modes using an integrated dielectric metasurface. *Photonics Research* **8**, 564-576 (2020).

3 Nanfang, Y., Genevet, P., Aieta, F., Kats, M. A., Blanchard, R., Aoust, G. *et al.* Flat Optics: Controlling Wavefronts With Optical Antenna Metasurfaces. *IEEE Journal of Selected Topics in Quantum Electronics* **19**, 4700423-4700423 (2013).

4 Balthasar Mueller, J. P., Rubin, N. A., Devlin, R. C., Groever, B. & Capasso, F. Metasurface Polarization Optics: Independent Phase Control of Arbitrary Orthogonal States of Polarization. *Physical Review Letters* **118**, 113901 (2017).
